# The impact of negative emotions on adolescents’ nonsuicidal self-injury thoughts: an integrated application of machine learning and multilevel logistic models

**DOI:** 10.1371/journal.pone.0320104

**Published:** 2025-11-25

**Authors:** Chan-Young Ahn, Jin-Ha Kim, Sojung Kim, Jae-Won Kim, Jung-Jo Na, Dong Gi Seo, Jong-Sun Lee

**Affiliations:** 1 Department of Psychology, Kangwon National University, Chuncheon, Republic of Korea; 2 Department of Psychology, Hallym University, Chuncheon, Republic of Korea; 3 Department of Psychology, Yeungnam University, Gyeongsan, Republic of Korea; 4 Division of Child and Adolescent Psychiatry, Department of Psychiatry, Seoul National University Hospital, Seoul, Republic of Korea; 5 Department of IT Media Engineering, Duksung women’s University, Seoul, Republic of Korea; Columbia University, UNITED STATES OF AMERICA

## Abstract

Non-Suicidal Self-Injury (NSSI) is a prevalent and complex behavior among adolescents, often linked to negative emotions such as loneliness, anxiety, and emptiness. Traditional self-report and experimental methods rely on autobiographical recall and are therefore vulnerable to bias and low ecological validity. Accordingly, approaches that repeatedly sample NSSI-related feelings and contexts in daily life such as Ecological Momentary Assessment (EMA) are needed. This study aimed to identify emotional predictors of NSSI thoughts among adolescents using machine learning and multilevel logistic regression. The study included 42 adolescents (aged 12–15 years) who had engaged in NSSI in the past year. Participants reported their mood and NSSI behaviors three times daily over a 14-day EMA period via a smartphone application. Predictor variables included depression, anxiety, loneliness, self-anger, anger towards others, shame, and emptiness. A random forest model identified loneliness (feature importance: 0.40), anxiety (0.18), and emptiness (0.14) as the most significant predictors of NSSI thoughts. Multilevel logistic regression confirmed these findings, showing that each one-unit increase in anxiety, loneliness, and emptiness corresponded to a 24%, 19%, and 24% increase in the odds of experiencing NSSI thoughts, respectively. The ICC value of 0.26 indicated substantial between-individual variance, justifying multilevel modeling. However, random effects analysis revealed no significant individual differences, suggesting uniform effects across participants. These findings highlight loneliness as the most influential predictor, emphasizing the need to address social connections in interventions. Combining machine learning with traditional statistical methods enhanced interpretability, providing practical insights for developing tailored, emotion-focused interventions for adolescents engaging in NSSI.

## Introduction

Nonsuicidal Self-Injury (NSSI) is defined as the deliberate and direct harming of one’s own body tissues without the intent to die [1]. Specifically, cutting the body with a sharp instrument is a common attempt, and other self-injurious behaviors, such as burning or intentionally hitting oneself, occur in a variety of ways and intensities [2,3]. According to the diagnostic criteria proposed in the DSM-5 (Fifth Edition of the Diagnostic and Statistical Manual of Mental Disorders), NSSI applies to individuals who have engaged in deliberate self-harm without suicidal intent on 5 or more days in the past year [4]. NSSI typically begins in early adolescence and may persist for several years [4]. A meta-analysis study on the prevalence of NSSI [5] found that prevalence peaked in adolescence at 17.2%, followed by 13.4% in early adulthood and 5.5% in adulthood, with a gradual decline in prevalence after adolescence. Another study found that globally, the lifetime prevalence of self-injurious behavior among adolescents is 19% and 20% for non-suicidal self-injury [6]. However, the actual prevalence of NSSI is likely to be higher than this, given that self-injurious behaviors are often secretive and private due to social stigma and concerns about being seen, and no specific medical attention is sought [7,8].

Self-injurious behavior is highly associated with several psychiatric symptoms. Specifically, depressive and anxiety disorders and borderline personality disorder are highly associated with NSSI [2,9,10], as are eating disorders and posttraumatic stress disorder [2,11,12]. It has also been found to be associated with a variety of psychiatric disorders, including attention deficit hyperactivity disorder, bipolar disorder, and substance use problems [9,10,13,14]. In particular, a serious problem with self-injurious behavior is its high association with suicide. Several studies have shown that, paradoxically, non-suicidal self-injury is a predictor of future suicidal behavior [15,16], and actual suicide attempt intent reported in 70.8% of adolescents who engage in self-injury repeatedly [17]. An individual’s ability to directly carry out a suicide is referred to as suicidal capability [18], and repeated self-injurious behaviors are quite dangerous in that they increase suicidal capability by decreasing fear of suicide, which ultimately increases the risk of suicide [18,19]. Repeated self-injurious behaviors during adolescence can make it difficult to succeed in school, career, and interpersonal relationships, which are key developmental tasks in early adulthood [20].

A growing body of research has identified factors that contribute to the development and maintenance of NSSI, aligning with several theoretical models [1,3,21]. Collectively, the results suggest that one of the most common reasons for engaging in self-injurious behavior is to avoid or regulate negative emotions. Negative emotions that have been reported to play a role in NSSI include depression, anxiety, loneliness, anger toward self or others, rejection, and guilt [22–26]. Individuals who report NSSI experience high levels of negative emotions [27], with some research utilizing longitudinal designs showing that negative emotions increase prior to the self-injurious behavior and decrease in the hours following the self-injurious behavior [12,22]. However, the alleviation of negative emotions caused by self-injurious behaviors is temporary and does not address the underlying issue, leading to the recurrence of such behaviors.

The nature of NSSI and its associated emotions changes over time and across contexts, making it difficult to measure with one-time data collection. Traditional self-report and experimental studies relying on autobiographical memory are prone to biases, limiting ecological validity [28]. Ecological Momentary Assessment (EMA) repeatedly records individuals’ real-time experiences, allowing for the measurement of changes in behavior and understanding of situational effects with high ecological validity and minimal recall bias [29]. Studies show that EMA is effective for exploring NSSI even with small samples [30,31].

Studies exploring risk factors for NSSI using EMA have been thus increasing, but their predictive power remains weak [32,33]. This limitation appears to be partly due to an over-reliance on traditional statistical methodologies [33]. Traditional approaches impose linearity on relationships and often limit the number of predictors that can be examined simultaneously, forcing researchers to rely on overly simplified models [36,37]. To complement these limitations, this study incorporated a machine learning technique—random forest—primarily as an exploratory tool for identifying the relative importance of multiple predictors in a data-driven manner.

In investigating NSSI, there is a growing trend towards actively utilizing machine learning approaches such as logistic regression and random forest [34,35]. According to a systematic review study, machine learning techniques have demonstrated higher predictive accuracy for self-injurious thoughts and behaviors (SITB) compared to traditional statistical methods [36]. This suggests that machine learning methods can be reliably used for variable selection concerning SITB risk factors. Notably, random forest provides advantages such as robustness against overfitting, the ability to evaluate multiple correlated predictors simultaneously, and the provision of feature importance indices that highlight the most influential variables. These characteristics are particularly useful when exploring psychological constructs such as negative emotions, which are conceptually and statistically interrelated. The use of machine learning for exploratory analysis can uncover hidden patterns and generate hypotheses, guiding further research and theory development [37,38].

However, despite these strengths, such methods are often challenging to apply in confirmatory research due to their low interpretability. To address this limitation, this study complemented machine learning techniques with logistic regression analysis to enhance interpretability by allowing researchers to directly select and examine variables of interest [39]. Finally, the aim of this study is to explore key emotional predictors of NSSI thoughts in adolescents by employing machine learning techniques alongside multilevel logistic regression analysis.

## Materials and methods

### Participants

This study included 42 adolescents aged 12–15 years. Inclusion criteria required that both the adolescents and their legal guardians voluntarily agreed to participate in the study and that the adolescents had engaged in NSSI on one or more days within the past year, with a minimum total frequency of at least one episode. Past-year NSSI frequency was assessed using the Self-Harm Screening Inventory (SHSI), and parental consent was obtained along with the adolescent’s own consent prior to enrollment. Participants were recruited on a nationwide scale from multiple elementary and middle schools in South Korea.

Exclusion criteria were as follows: cases where either the adolescent or their legal guardian did not provide consent; responses with missing data or patterns indicating random responding (e.g., identical responses across items); lack of access to a personal smartphone; discontinuation before completion; or current receipt of psychotherapy or counseling. In addition, participants were excluded if they presented with psychotic or manic symptoms, substance use disorders, or suicidal ideation and plans at a level requiring immediate intervention. The presence of recent suicidal intent or behavior was assessed using the Columbia Suicide Severity Rating Scale (C-SSRS).

### Procedure

Participant recruitment was conducted through DataSpring, a professional research firm, in accordance with predetermined eligibility criteria. Two rounds of panel-wide invitations were sent to registered panel members (Round 1: 18,043; Round 2: 5,171), yielding 3,123 respondents to the online screening questionnaire (Round 1: 2,225; Round 2: 898). The invitations and the screening survey were administered by DataSpring; the research team received the screening survey data and the contact information for adolescents who met eligibility criteria. Respondents were provided detailed information about the study (purpose, procedures, potential risks/benefits) and informed of their right to withdraw at any time without consequences. Based on the screener, 111 adolescents met eligibility criteria (Round 1: 92; Round 2: 19). Of these, 63 could not be contacted or declined final participation (Round 1: 47; Round 2: 16), resulting in 48 enrolled adolescents. Legal guardians provided informed consent, and adolescents provided assent electronically via the study’s online survey form, as approved by the Institutional Review Board of Kangwon National University (KWNUIRB-2022-01-009-001). Recruitment occurred from May 25, 2022, to June 4, 2022.

The final sample in the analysis comprised 42 participants, after excluding one participant who withdrew during the study and five participants who failed to meet the minimum 50% ecological momentary assessment (EMA) response rate criterion. This systematic screening and enrollment process ensured the quality and reliability of the collected data while maintaining ethical standards for research with adolescent participants.

Upon the completion of the informed consent process, participants were integrated into the study through the utilization of the dedicated application, Mind Care, purposefully developed for this research endeavor. Subsequently, participants underwent a preassessment, involving the completion of a comprehensive questionnaire encompassing the Columbia Suicide Severity Rating Scale (C-SSRS), Center for Epidemiological Studies Depression Scale for Children (CES-DC), and Revised Children’s Manifest Anxiety Scale (RCMAS).

Throughout a 14-day EMA period, participants utilized a smartphone app to report their mood three times daily and document instances of NSSI behaviors and the intensity of NSSI thoughts. This resulted in a cumulative total of 41 assessments over the specified timeframe, with participants engaging in assessments three times a day for 13 days and twice a day on the concluding day.

EMA assessments were delivered via the study smartphone application on fixed time windows three times per day. An initial push notification was sent at 12:00 (afternoon), 18:00 (evening), and 21:00 (night). For non-responders, the app issued up to two reminders within each window at +30 minutes and +60 minutes after the initial notification. To accommodate middle-school schedules, responses were accepted only within the following predefined windows: 12:00–17:59 (afternoon), 18:00–20:59 (evening), and 21:00–23:59 (night). Thus, the minimum spacing between initial prompts was 6 hours (12:00 → 18:00) and 3 hours (18:00 → 21:00), whereas reminder notifications within a window were spaced 30 minutes apart. Responses submitted outside the designated windows were not accepted for that epoch. Examples of the EMA questionnaires used in this study are provided in Appendix 2 in S1 Appendix.

Following the completion of the 14-day EMA period, participants were subjected to a postassessment utilizing the same questionnaire employed during the preassessment phase.

### Measures

#### Center for Epidemiological Studies Depression Scale for Children (CES-DC).

Developed by Weissman et al. (1980) [40], the Depression Scale for Children is a scale used to measure depression in adolescents. It consists of 20 items and measures depressive mood over the past week on a 4-point Likert scale (0: not at all, 3: very much). The Cronbach’s alpha in this study was.828.

#### Revised Children’s Manifest Anxiety Scale (RCMAS).

The Revised Children’s Manifest Anxiety Scale developed by Reynolds and Paget [41] was used to assess the level of anxiety in adolescents. The RCMAS is designed to measure the presence of various anxiety-related symptoms and is applicable to adolescents. It consists of 37 items with a 2-point Likert scale of “yes” (1 point) or “no” (0 point) for each item. The Cronbach’s alpha in this study was.823.

#### The Self-Harm Screening Inventory (SHSI).

The Self-Harm Screening Inventory (SHSI) is a brief self-report questionnaire designed to screen adolescents for self-harm behavior [42]. It consists of 10 binary items (yes/no) that inquire about self-harm behavior over the past year. In this study, self-harm behavior was assessed using a 6-point Likert scale (0: Not applicable/ 1: Once/ 2: 2–5 times/ 3: 6–10 times/ 4: 11–30 times/ 5: More than 31 times) to capture the frequency and types of self-harm, as well as to evaluate whether medical treatment was sought. The Cronbach’s alpha in this study was.916.

#### Columbia Suicide Severity Rating Scale (C-SSRS).

The Columbia Suicide Severity Rating Scale (C-SSRS) was developed by Posner and Brent [43] to assess the severity of and changes in suicidal ideation and behavior. It is designed to measure four components: the severity of suicidal ideation, the intensity of suicidal ideation, suicidal behavior, and lethality. Additionally, it accommodates different assessment periods based on research or clinical needs. In this study, one item assessing active suicidal ideation with some intent to act, without specific plan, and another item assessing active suicidal ideation with specific plan and intent were used to measure the presence or absence of suicidal intent and attempts over the past month, using dichotomous “yes” or “no” responses.

#### Self-injury and suicidality questionnaire.

The Self-Injury and Suicidality Questionnaire was delivered via a smartphone-based EMA app. It consisted of binary items (yes/no) assessing the presence of self-injurious and suicidal thoughts, as well as self-injurious and suicidal behaviors, since the previous EMA session. If participants reported engaging in self-injurious behavior since the last assessment, additional questions were presented to assess (a) whether they experienced suicidal thoughts during or immediately before the self-injury (0: Not at all ~ 3: I thought very seriously about death and wanted to die), and (b) what specific methods were used for self-injury (e.g., cutting, hitting, or burning).

#### Mood appraisal questionnaire.

The Mood Appraisal Questionnaire was delivered via a smartphone-baseed EMA app. Participants responded their emotions they are experiencing at that moment on a VAS 3 times a day. The mood Visual Analogue Scale (VAS) consisted of a horizontal line from “Not at all” at one end to “Extremely” at the other. Specifically, participants are instructed to respond to their feelings of depression, anxiety, shame, anger towards myself, anger towards others, loneliness, and emptiness on an 9-point Likert scale. Within-person reliability of these repeated emotion ratings was excellent (ICC = .977, 95% CI [.975,.978]), indicating high consistency across assessments.

### Data analysis

This study aims to elucidate the causal factors behind thoughts of non-suicidal self-injury (NSSI). To achieve this, we employed a mixed-methods approach, focusing initially on quantitative analysis followed by a multilevel logistic regression to account for the nested structure of the data. In addition to multilevel logistic regression, which served as our primary confirmatory analytic approach, we applied a random forest model in an exploratory manner. Random forest was selected because it can simultaneously handle multiple correlated predictors, provides unbiased feature importance indices for identifying the most influential variables, and reduces overfitting through ensemble learning. These characteristics are particularly valuable when exploring psychological constructs such as negative emotions that are conceptually and statistically interrelated. In this study, the random forest results guided the identification of key emotional predictors, which were subsequently tested and confirmed using multilevel logistic regression within the nested EMA design.

The initial quantitative analysis was conducted using a random forest algorithm. Random forest, developed by Breiman [44], builds upon the decision tree classifier, which determines optimal split variables and points at each node using impurity measures such as chi-square statistics, Gini index, and entropy [45]. While decision trees are more interpretable than other supervised learning techniques, they are often less predictive and can be highly sensitive to data variations [46]. Random forest addresses these shortcomings by incorporating bootstrap aggregation and randomization of variables to enhance randomness and robustness. By randomly selecting subsets of variables, it generates diverse decision trees, reducing their correlation and improving prediction accuracy [47]. This method was chosen for its robustness in handling a large number of predictor variables and for its utility in feature importance evaluation. The dependent variable in this analysis was the occurrence of NSSI thoughts, coded as a binary outcome (0 for absence, 1 for presence). Predictor variables included measures of depression, anxiety, loneliness, self-anger, anger towards others, shame, and emptiness.

For the random forest analysis, the unit of analysis was the observation (epoch), yielding a total of 1,416 EMA observations across 42 participants, with 7 predictor variables. Approximately 21% of these observations indicated the presence of NSSI thoughts (303/1,416). With a relatively small set of predictors and over 1,000 observations, random forest training with 5-fold cross-validation provides stable and robust estimation with low variance [44,48]. Random forest model was configured with a maximum depth of three for each decision tree, ensuring that the model was sufficiently complex to capture relevant patterns in the data without overfitting. The ensemble consisted of 1,000 trees, providing a robust aggregation of decisions from multiple trees to improve the overall predictive accuracy and stability of the model. For model validation, a 5-fold cross-validation approach was employed.

Through the random forest analysis, we calculated the feature importance scores to identify which variables most significantly influenced the decision-making process in the algorithm. Feature importance values were computed using the mean decrease in impurity (MDI) criterion implemented in the scikit-learn RandomForestClassifier [49]. Specifically, each split in the decision trees reduces the Gini impurity of the classification. The magnitude of this impurity reduction is attributed to the variable used for the split, and the values are then averaged across all trees in the forest and normalized to sum to 1. The resulting feature importance score reflects the relative contribution of each predictor to improving the purity of the classification, with higher scores indicating greater influence on the model’s predictions [50].

However, it is important to note that feature importance values have limitations. First, they reflect only the relative contribution of predictors within the fitted model and do not indicate causal effects, effect sizes, or directions of association. Second, when predictors are correlated, importance scores may be biased or distributed across related variables, making interpretation less straightforward. Finally, feature importance values are sample- and model-dependent, meaning that they can vary across different datasets or model specifications. For these reasons, we used random forest primarily as an exploratory tool for variable importance, and complemented it with multilevel logistic regression to provide statistically interpretable estimates (e.g., odds ratios, confidence intervals) suitable for confirmatory analysis.

Furthermore, the random forest analysis did not account for the multilevel structure of the data. Each participant provided multiple observations, creating a nested data format that the random forest model does not inherently consider. In social science research, an Intraclass Correlation Coefficient (ICC) value greater than 0.20 typically necessitates a multilevel analysis approach [51].

To address this limitation, we conducted a multilevel logistic regression analysis including fixed and random effects of predictors [52]. The multilevel logistic regression, also known hierarchical logistic regression model, is particularly suited for analyzing data with a nested structure. This model helps capture the variability at both the within and between levels, offering a comprehensive understanding of how predictors at different levels influence the binary outcome. The dependent variable remained the binary indicator of NSSI thought occurrence. Given that our predictors are all related to negative emotional states and share considerable covariates, we carefully selected variables for inclusion in the regression model. Instead of including all predictors simultaneously, which could lead to multicollinearity issues, each predictor was included in separate models [53]. This approach allowed us to isolate the effect of each predictor on NSSI thoughts while controlling for the nested data structure. For the multilevel logistic regression models, our dataset comprised Level-1 = 1,416 repeated observations nested within Level-2 = 42 participants. Because methodological guidelines indicate that about 30–50 clusters are generally sufficient to obtain unbiased fixed-effect estimates in multilevel logistic regression [54], our sample of 42 clusters meets these recommendations. By contrast, reliable estimation of random-slope or more complex random-effect structures typically requires much larger Level-2 samples. Accordingly, we focused on fixed effects and analyzed each emotional predictor in a separate model to minimize over-parameterization.

The analysis using Random Forest was conducted with Python version 3.10.12. To estimate the multilevel logistic regression model, Bayesian estimator in Mplus 8.3 was used.

## Results

### Demographic and clinical characteristics

Before conducting the main analysis, a descriptive statistical analysis was performed to examine the demographic characteristics of the participants (Table 1). Among the participants, 64.3% were female (*n* = 27) and 35.7% were male (*n* = 15). Participants’ age ranged from 12 to 15 years (M = 14.71, SD = 0.64). The majority were 15 years old (third-year middle school students) (78.6%, *n* = 33), followed by 14 years old (second-year middle school students) (16.7%, *n* = 7), with only one participant aged 12 years (first-year middle school student) (2.4%). This imbalance was also reflected in the elevated kurtosis value (8.00), suggesting a restricted range and a peaked distribution. Regarding the participants’ self-perceived household economic status, the middle-class category represented the largest proportion (45.2%, *n* = 19), followed by upper-middle (33.3%, *n* = 14), lower-middle (16.7%, *n* = 7), and low (4.8%, *n* = 2). Regarding household size, four-person households were most common (45.2%, *n* = 19), followed by three-person households (33.3%, *n* = 14), five-person households (16.7%, *n* = 7), and six-person households (4.8%, *n* = 2).

**Table 1:** Descriptive statistics of demographic and clinical characteristics.

	Variable	M (SD)	Min-Max	Kurtosis	Skewness
Demographic Information	Sex	15:27		−1.70	−.62
	Economic status	14:19:7:2		.00	.66
	Age	14.71 (0.64)	12-15	8.00	−2.67
Clinical Characteristics (Pre-assessment)	CES-DC	24.24 (11.50)	3-44	−1.26	−.08
	RCMAS	54.45 (6.84)	39-69	−.35	.13
	SHSI	5.52 (7.22)	1-37	9.03	2.86
Clinical Characteristics (EMA – 14 days)	Depression	2.49 (1.49)	1-7.3		
	Anxiety	2.46 (1.35)	1-7.05		
	Loneliness	2.42 (1.71)	1-7.54		
	Anger towards self	2.45 (1.54)	1-7.12		
	Anger towards others	2.02 (1.16)	1-5.68		
	Shame	1.93 (1.27)	1-6		
	Emptiness	2.33 (1.51)	1-6.61		
	NSSI thoughts^a^	1.21 (2.17)	0-10		
	Suicidal thoughts^a^	1.19 (4.37)	0-26		

Note. Sex (Male:Female), Economic status (Upper-middle:Middle:Lower-middle:Low).

CES-DC = Center for Epidemiological Studies Depression Scale for Children; RCMAS = Revised Children’s Manifest Anxiety Scale; SHSI = Self-Harm Screening Inventory; EMA = ecological momentary assessment.

^a^NSSI thoughts and Suicidal thoughts were assessed via EMA as binary items (1 = present, 0 = absent), with reported values reflecting the mean number of occurrences across the 41 EMA prompts.

To examine the correspondence between pre-assessment self-report measures and EMA measures, correlation analyses were conducted (Table 2). EMA-rated depression was positively associated with CES-DC scores (*r* = .47, *p* < .01), indicating that participants who reported higher depressive symptoms in the baseline assessment also tended to report greater momentary depression during the 14-day EMA period. Similarly, EMA-reported NSSI thoughts were positively correlated with SHSI scores (ρ = .39, *p* < .05), suggesting that adolescents who endorsed more frequent self-harm behavior in the pre-assessment were more likely to report NSSI thoughts in daily life.

**Table 2:** Associations between pre-assessment self-report measures and EMA (14-day) ratings.

Variable	Pre-assessment measure	EMA (14-day)	Association
Depression	CES-DC	EMA-rated depression	Pearson *r* = .47, *p* < .01
NSSI	SHSI	EMA-reported NSSI thoughts occurrence	Spearman ρ =.39, *p* <.05

*Note.* CES-DC = Center for Epidemiological Studies Depression Scale for Children; SHSI = Self-Harm Screening Inventory; EMA = ecological momentary assessment. Pearson correlations were used for continuous variables (CES-DC and EMA depression ratings). Spearman rank correlations were used when comparing ordinal/scale measures with binary EMA measures (SHSI and EMA NSSI).

### Random forest

The average EMA response rate was 86.5%. The confusion matrix, shown in Table 3, provides a comprehensive overview of the model’s performance. Using formulas 1.1 to 1.5, we derive the following evaluation metrics for the model: The accuracy of the model is 0.792, calculated as the ratio of true positives and true negatives to the total number of instances. However, relying solely on accuracy can be misleading when assessing the model’s performance, especially in the presence of class imbalance. Therefore, additional metrics are considered to provide a more detailed evaluation.

**Table 3:** Confusion Matrix for Binary Classification.

		Predicted	
		0	1
Actual	0	True Negative(TN)	False Positive(FP)
		810	303
	1	False Negative(FN)	True Positive(TP)
		189	114

The sensitivity, or recall, is 0.376, indicating that the model correctly identifies 37.6% of the actual positive cases. Specificity, which measures the model’s ability to correctly identify negative cases, is 0.728. The positive predictive value (PPV), representing the proportion of positive results that are true positives, is 0.273. Conversely, the negative predictive value (NPV), which measures the proportion of negative results that are true negatives, is relatively high at 0.811.

This data suggests that while the model performs well in identifying negative instances, its sensitivity is low due to the zero-inflated nature of the dataset, where instances of class 0 make up 21.3% of the total data. Consequently, the model exhibits a higher NPV because it accurately predicts the abundant negative instances. However, the PPV is low, reflecting the difficulty in correctly predicting positive instances. Despite the lower PPV of 0.273, this value is considered relatively high compared to other studies, indicating that the model performs reasonably well in identifying positive cases [55].

**Fig 1:**
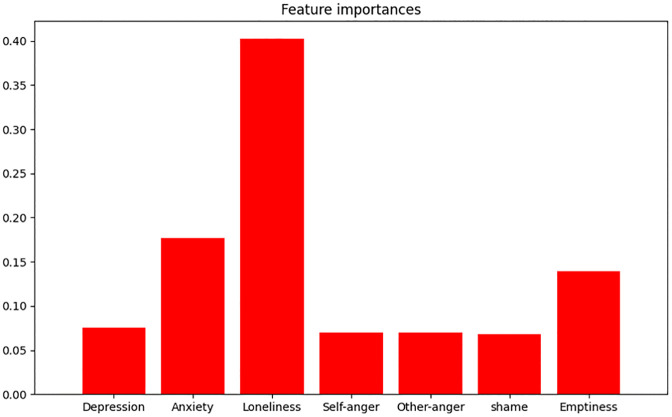
Feature importance scores of the predictors.


The feature importance of the random forest is viewed in Fig 1. The y-axis in Fig 1 represents the feature importance scores generated by the random forest model. These scores indicate the relative importance of each predictor variable in influencing the model’s predictions. Feature importance in a random forest is determined based on how much each feature decreases the impurity of the split, which in this case is measured using the Gini impurity for classification problems. Among all variables, loneliness had the highest score of 0.40. Anxiety ranked as the second highest among all variables with a score of 0.18, followed by emptiness, which had a score of 0.14. Depression, self-anger, anger towards others, and shame showed similar levels of feature importance scores in the analysis (Depression = 0.08, self-anger = 0.07, anger towards others = 0.07, shame = 0.07).

$$\text{Accuracy}\;=\;\frac{TP+TN}{TP+FN+FP+TN}=\;\text{0}.\text{792}$$ (1.1)

$$\text{Sensitivity}\;=\;\frac{TP}{TP+FP}=\;\text{0}.\text{376}$$ (1.2)

$$\text{Specificity}\;=\;\frac{TN}{TN+FP}=\;\text{0}.\text{728}$$ (1.3)

$$\text{PPV}\;=\;\frac{TP}{TP+FP}=\;\text{0}.\text{273}$$ (1.4)

$$\text{NPV}\;=\;\frac{TN}{TN+FN}=\;\text{0}.\text{811}$$ (1.5)

Note. TN (True Negative) = correctly identified negative cases; FP (False Positive) = negative cases incorrectly classified as positive; FN (False Negative) = positive cases incorrectly classified as negative; TP (True Positive) = correctly identified positive cases.

### Multilevel logistic regression

The ICC (Intraclass Correlation Coefficient) value for the dependent variable ‘occurrence of NSSI thoughts’ collected in the research was found to be 0.26. An ICC value of 0.26 indicated that the between level variance accounted for approximately 26% of the total variance in the dependent variable. This suggests the need for employing a multilevel model in analyzing this data. Table 4 displays the result of a multilevel logistic regression model that predicts NSSI thought with depression, anxiety, loneliness, self-anger, anger towards others, shame, and emptiness as predictor variables. The detailed equations of the multilevel logistic regression models are presented in Appendix 1 in S1 Appendix. In the fixed effects analysis, the variables anxiety, loneliness, and emptiness were found to be significant [anxiety (0.216, *p* = 0.005), loneliness (0.177, *p* = 0.013), emptiness (0.217, *p* = 0.027)]. For each one-unit increase in anxiety, there is a 24% increase in the odds of experiencing an NSSI thoughts [exp(.216) = 1.24]. For each additional unit increase in loneliness, the odds of NSSI thoughts increase by 19% [exp(.177) = 1.19]. An increase in the measure of emptiness by one unit is associated with a 24% increase in the odds of experiencing NSSI thoughts [exp(.217) = 1.24]. It is noteworthy that the outcomes of the random forest’s feature importance scores and the multilevel logistic regression analysis are in concordance. In the random effects analysis, none of the variables were found to be significant. This suggests that there were no individual differences in the impact of these variables on NSSI thoughts, indicating a uniform influence of these variables across individuals.

**Table 4:** Multilevel logistic regression models with each negative emotions as a predictor.

	Effects	Estimate	S.E	p-value
Depression	Fixed effect($\gamma_{\text{00}}$)	0.196	0.127	.123
	Random effect($\tau_{\text{00}}$)	0.044	0.062	.475
Anxiety	Fixed effect($\gamma_{\text{10}}$)	0.216	0.077	.005*
	Random effect($\tau_{\text{10}}$)	0.000	0.000	.634
Loneliness	Fixed effect($\gamma_{\text{20}}$)	0.177	0.071	.013*
	Random effect($\tau_{\text{20}}$)	0.000	0.000	.500
Self-anger	Fixed effect($\gamma_{\text{30}}$)	0.117	0.149	.432
	Random effect($\tau_{\text{30}}$)	0.040	0.048	.404
Anger towards others	Fixed effect($\gamma_{\text{40}}$)	−0.056	0.168	.738
	Random effect($\tau_{\text{40}}$)	0.198	0.134	.141
Shame	Fixed effect($\gamma_{\text{50}}$)	−0.270	0.392	.490
	Random effect($\tau_{\text{50}}$)	0.463	0.683	.498
Emptiness	Fixed effect($\gamma_{\text{60}}$)	0.217	0.099	.027*
	Random effect($\tau_{\text{60}}$)	0.001	0.011	.921

Note. An asterisk (*) indicates that the p-value is less than.05.

## Discussion

The present study aimed to identify negative emotions that predict NSSI thoughts using random forest techniques and multilevel logistic regression. The key findings show that loneliness emerged as the most influential variable, with a feature importance score of 0.40, followed by anxiety and emptiness. These results were also confirmed by multilevel logistic results. These findings underscore the substantial impact of loneliness on adolescents’ NSSI thoughts, aligning with prior evidence that loneliness is a key factor in adolescent NSSI [26,56].

Adolescence is a critical period marked by evolving social connections and a heightened need for intimacy, factors that contribute to the persistence of loneliness [57]. This stage is crucial for understanding the dynamics between loneliness and NSSI, which can be conceptualized within the interpersonal functioning model of NSSI [1]. These behaviors can serve not only as a means to alleviate emotional distress but also play a complex role in social interactions [58]. Specifically, NSSI may function as a cry for help or a method to gain support, thereby strengthening social bonds. This social reinforcement can inadvertently maintain and even reinforce the cycle of self-injurious behavior, as suggested by Nock and Prinstein [59].

Furthermore, according to the interpersonal theory of suicide, thwarted belongingness, a feeling stemming from loneliness or isolation, substantially elevates an individual’s risk of suicide [18,60]. Indeed, the interplay between NSSI and suicidal thoughts and behaviors has been documented, showing a close associations, which suggests that underlying interpersonal states fostered by loneliness are critical contributors to both NSSI and suicidal behaviors [61].

Our study shows emptiness is one of the important factors we need to note in accounting for adolescents’ self-injury. While research examining the direct relationship between emptiness and NSSI has been limited, existing evidence suggests that emptiness may lead individuals to engage in NSSI as a means to enhance emotional and sensory experiences [62]. Prior research has demonstrated that emptiness is significantly associated with suicidal ideation and attempts [63]. In a study of women diagnosed with borderline personality disorder (BPD), feelings of emptiness preceded NSSI, suggesting that NSSI may serve as a coping mechanism for negative emotional states, particularly emptiness [64]. Furthermore, research involving college students who engaged in NSSI, 67% reported that feelings of emptiness sometimes preceded their NSSI behaviors [63]. Other studies involving BPD populations have shown that, compared to impulsivity, affective instability, and anger, emptiness is the only BPD symptom that predicts all eight indicators of psychosocial morbidity, including suicidality [65]. Moreover, individuals who experience maltreatment during childhood are at increased risk of experiencing emptiness during adolescence and adulthood. This psychological condition is theorized to increase the likelihood of engaging in NSSI as a means of alleviating feelings of emptiness and achieving a sense of well-being [66,67]. The present findings align with and extend these previous studies.

NSSI is associated with anxiety, which is considered a significant trigger for these behaviors [68,69]. For example, NSSI is often utilized as a method to alleviate distress related to anxiety symptoms [70], and anxiety reduction is frequently reported as one of the primary functions of NSSI [71]. Additionally, individuals who meet the diagnostic criteria for an anxiety disorder—characterized by frequent and intense negative emotions and efforts to avoid or escape such experiences—report higher rates of NSSI compared to those without an anxiety disorder diagnosis [72]. Furthermore, a positive association between anxiety and NSSI has also been observed in nonclinical community samples [73]. Consistent with these previous findings, our results identified anxiety as a key factor of NSSI among adolescents, further supporting the crucial role of anxiety in understanding and addressing adolescents’ self-harming behaviors.

The relationship between NSSI and anxiety can be understood within the framework of one of the widely accepted theories, the emotional cascade theory [74]. This theory suggests that even minor emotional stimuli can trigger repetitive negative thinking (e.g., rumination), which amplifies the experience of negative emotions and leads to a vicious cycle, or an emotional cascade [75]. In an attempt to halt this emotional cascade, individuals may resort to extreme forms of distraction, such as NSSI. Following this, they often experience a sense of relief due to negative reinforcement, which is analogous to the relief experienced after avoidance in the context of anxiety.

Given these insights, it is imperative for clinicians to design targeted interventions that address loneliness when developing strategies for adolescents engaging in self-injury. Such interventions could focus on enhancing social connectedness by fostering positive and supportive relationships with family, friends, and peers. In addition, efforts may be necessary to disseminate and promote psychoeducational manuals on emotional regulation strategies aimed at reducing loneliness in self-injuring adolescents, targeting teachers and clinicians as key disseminators [76].

For anxiety reduction, cognitive-behavioral strategies—particularly exposure (situational and interoceptive), modification of safety behaviors, intolerance-of-uncertainty work, skills for urge management (e.g., stimulus control, alternative behaviors), and foundations such as sleep hygiene—may decrease reliance on NSSI for short-term relief. For emptiness, DBT skills (mindfulness, emotion regulation, distress tolerance, interpersonal effectiveness) alongside values/identity work, behavioral activation, and sensory-modulation strategies can help increase engagement with rewarding activities and reduce the drive to use NSSI to “feel something.”

The Intraclass Correlation Coefficient (ICC) value of 0.26 in the present study indicates that 26% of the variance in NSSI thoughts can be attributed to between-individual differences. This justified the utilization of a multilevel model, allowing for an in-depth examination of the impact of various predictors. The results of the fixed effects analysis highlighted the significance of anxiety, loneliness, and emptiness. Each unit increase in anxiety, loneliness, and emptiness corresponded to a 24%, 19%, and 24% increase in the odds of experiencing NSSI thoughts, respectively. These findings align with the random forest results, reinforcing the robustness and consistency of the identified predictors.

The noteworthy concordance between the random forest’s feature importance scores and the results of the multilevel logistic regression analysis enhances the reliability of the identified predictors. Both approaches underscored the pivotal role of loneliness, anxiety, and emptiness in influencing NSSI thoughts. This convergence strengthens the validity of the study’s findings, demonstrating a consistent pattern across different analytical methodologies. Furthermore, this suggests that machine learning techniques such as random forest can be reliably applied to explore predictive factors that explain the characteristics of non-suicidal self-injury [36,38].

In contrast, the random effects analysis revealed no significant individual differences, suggesting a uniform impact of the identified variables. This finding implies that, while the factors influencing NSSI thoughts are consistent, their intensity may vary among individuals. The absence of significant random effects highlights the universal relevance of loneliness, anxiety, and emptiness in shaping NSSI thoughts, irrespective of individual differences.

The strength of this study lies in its ability to capture fluctuations in emotions and self-injurious thoughts experienced by individuals in their daily lives through the use of digital-based methodologies. A key advantage of our design is that, by using digital EMA with repeated measurements, we collected adolescents’ emotions and NSSI thoughts in their daily lives with reduced recall bias and high ecological validity. Variables related to NSSI, including emotions, exhibit fluctuations within a day [77]. Therefore, traditional retrospective self-report methods may fail to adequately capture the emotional dynamics that change within minutes or hours in daily life. Thus, researchers need to devise meticulous and refined approaches for longitudinal and real-time data collection to assess self-injurious behavior and emotions [78].

Methodologically, this study also benefits from employing sophisticated statistical approaches. In psychological research, particularly in studies of complex behaviors like NSSI, traditional statistical methods often face limitations due to the intricate interplay of numerous predictors. In our study, the integration of random forest analysis with multilevel logistic regression allowed us to robustly identify key predictors of NSSI thoughts, underscoring the potential of these advanced analytical approaches for gaining deeper insights into psychological phenomena and improving mental health outcomes.

Nonetheless, this study has several limitations. First, our machine learning model demonstrated higher accuracy in predicting true negatives than true positives, as reflected in its relatively low sensitivity (0.376) and positive predictive value (PPV = 0.273), but relatively high negative predictive value (NPV = 0.811). In other words, the model was more effective at identifying instances in which NSSI thoughts were absent rather than present. This pattern suggests that while loneliness and other negative emotions were useful indicators for ruling out NSSI thoughts, they were less effective in precisely predicting when NSSI thoughts would occur.

These results suggest that loneliness may be a necessary but not sufficient condition for the occurrence of NSSI. In other words, loneliness may serve as a moderator that distinguishes between individuals who experience loneliness and engage in NSSI and those who do not. This can be interpreted within the framework of the Interpersonal Theory of Suicide [18], which conceptualizes loneliness as one component of thwarted belongingness—a multidimensional construct reflecting an unmet need to belong [79]. Within this framework, loneliness may represent a necessary precondition for suicidal or self-injurious thoughts, but additional interpersonal and contextual factors likely moderate whether loneliness progresses into NSSI. For instance, adverse experiences such as family conflict, childhood abuse, or the absence of reciprocally caring relationships may exacerbate the effects of loneliness, whereas social support from family, peers, or teachers may buffer against it. Thus, one important direction for future research is to identify moderators that distinguish adolescents who experience loneliness but do not engage in NSSI from those for whom loneliness contributes to self-injurious thoughts. This approach may clarify why loneliness shows strong associations with NSSI at the group level, but not all lonely individuals are equally at risk.

Second, although the overall compliance rate in our study was high (average 86%), this may reflect a more motivated or organized subset of adolescents who were willing and able to complete repeated EMA prompts. While such adherence is comparable to or higher than that typically observed in adolescent EMA studies, it may limit the generalizability of our findings to youth who are less engaged, less organized, or at higher clinical risk. Our use of a two-week design, fixed prompt times, and reminder notifications likely helped maintain high compliance while minimizing participant burden. Future research should consider strategies to recruit and retain adolescents with lower expected adherence to ensure broader representativeness and enhance the external validity of EMA findings.

Third, the sex distribution of the sample (female = 27, 64.3%; male = 15, 35.7%), which may constrain generalizability given documented sex differences in the prevalence and expression of NSSI. Relatedly, we did not test sex (or other potential moderators) as interaction terms in our multilevel models, as the study was not designed or powered for stratified or cross-level moderation analyses. Future studies should recruit more balanced samples and sufficient Level-2 size to examine whether the identified emotional predictors of NSSI thoughts differ by sex or other clinically relevant moderators.

Fourth, we did not systematically assess broader clinical characteristics associated with NSSI, such as psychiatric diagnoses (e.g., borderline personality disorder), trauma history, or past treatment experiences. While depression levels were assessed and reported, the absence of other diagnostic and clinical information limited our ability to contextualize NSSI thoughts within participants’ broader psychopathology, and these variables could not be included as covariates in the present models. Future research should incorporate structured diagnostic assessments and measures of trauma and treatment history to determine whether the identified emotional predictors of NSSI thoughts remain robust when controlling for broader psychopathology.

Finally, the interest in applying machine learning approaches has grown across psychology and other research domains [80,81], these methods, despite their substantial advantages, also present notable challenges. In particular, random forest models have limited interpretability, making them less suitable as confirmatory tools in social science research where understanding the precise relationship between variables is crucial. This limited interpretability means that random forest primarily yields relative importance scores rather than interpretable parameters. As a result, it cannot directly provide effect sizes, directions of associations, or measures of statistical uncertainty such as odds ratios or confidence intervals [44]. These metrics are central in psychological and clinical research, where precise estimates are needed to evaluate theoretical models and to inform clinical decision-making. Without such information, the ability to translate predictive findings into practical or theoretical implications remains restricted.

This limitation is especially pertinent in psychology, where the ability to interpret and communicate findings is essential [45,82]. Therefore, it is important to complement machine learning methods with traditional statistical techniques, such as logistic regression, where researchers can directly select and examine variables of interest. These classical methods not only enhance interpretability but also allow for hypothesis-driven confirmatory analysis, ensuring that research findings are both robust and theoretically sound [39]. For this reason, in the present study, random forest was used in conjunction with multilevel logistic regression, with the former serving an exploratory role and the latter providing confirmatory evidence within the nested EMA design.

Building on these findings, future research should recruit larger and clinically diverse cohorts and explicitly test moderation and conditional effects—examining whether risk varies as a function of sex, social support, family conflict, trauma history, and related interpersonal/contextual factors. Designs that extend EMA with time-varying models (e.g., lagged multilevel models, VAR/DSEM) will allow tests of temporal precedence and moderated effects rather than cross-sectional associations. Methodologically, pairing EMA with digital phenotyping (passive indicators of sleep, activity, mobility, and social contact) and using explainable machine learning tools (permutation importance, SHAP) with class-imbalance–aware training (calibrated thresholds or cost weights) and out-of-sample validation will improve transparency and clinical utility.

On the intervention side, an important next step is to evaluate just-in-time adaptive interventions (JITAIs) that deliver brief, evidence-based supports precisely when risk increases, with modules that enhance belonging/reciprocal care and target anxiety/emptiness as indicated. Trials should compare moderator-guided, personalized targeting against one-size-fits-all approaches and assess feasibility and equity of delivery in school and clinical settings. These directions collectively move toward precise, scalable prevention and care for adolescents at risk of NSSI.

## Conclusions

This study’s findings, derived from both random forest analysis and multilevel logistic regression, consistently identify loneliness, anxiety, and emptiness as key predictors of NSSI thoughts. While machine learning methods proved valuable for exploratory analysis, their integration with traditional statistical approaches enhanced the robustness of our results. The findings have important clinical implications, suggesting that NSSI interventions should be tailored to address the unique negative emotions of individuals with NSSI, rather than applying a one-size-fits-all approach. This personalized approach, focusing on each individual’s unique combination of negative emotions, may lead to more effective treatment outcomes. This research not only validates the complementary use of different analytical methods in psychological research but also provides practical insights for developing targeted NSSI interventions.

## Supporting information

S1 Appendix Appendix 1. Detailed equations of the multilevel logistic regression models for adolescents’ nonsuicidal self-injury thoughts. Appendix 2. Examples of the EMA questionnaires used in this study. (DOCX)
