# Supplementary material for: The impact of negative emotions on adolescents’ nonsuicidal self-injury thoughts: an integrated application of machine learning and multilevel logistic models
Source: PLoS One. 2025 Nov 25;20(11):e0320104. doi: 10.1371/journal.pone.0320104 (PMC12646445; doi:10.1371/journal.pone.0320104)
Supplement: S1 Appendix — (DOCX) [file pone.0320104.s001.docx]

**Appendix 1. Detailed equations of the multilevel logistic regression models for adolescents’ nonsuicidal self-injury thoughts**

**Within-level**

| ${P_{ti}}_{({NSSI thought}_{ti} = 1 \vert{Depression}_{ti})}= \frac{1}{{1+e}^{-(\alpha_{0i}+\beta_{0i}{dep}_{ti})}}$ |
| --- |
| ${P_{ti}}_{({NSSI thought}_{ti} = 1 \vert{Anxiety}_{ti})}= \frac{1}{{1+e}^{-(\alpha_{1i}+\beta_{1i}{anx}_{ti})}}$ |
| ${P_{ti}}_{({NSSI thought}_{ti} = 1 \vert{Loneliness}_{ti})}= \frac{1}{{1+e}^{-(\alpha_{2i}+\beta_{2i}{lon}_{ti})}}$ |
| ${P_{ti}}_{({NSSI thought}_{ti} = 1 \vert{Self-anger}_{ti})}= \frac{1}{{1+e}^{-(\alpha_{3i}+\beta_{3i}{sel}_{ti})}}$ |
| ${P_{ti}}_{({NSSI thought}_{ti} = 1 \vert{Anger towards others}_{ti})}= \frac{1}{{1+e}^{-(\alpha_{4i}+\beta_{4i}{oth}_{ti})}}$ |
| ${P_{ti}}_{({NSSI thought}_{ti} = 1 \vert{Shame}_{ti})}= \frac{1}{{1+e}^{-(\alpha_{5i}+\beta_{5i}{sha}_{ti})}}$ |
| ${P_{ti}}_{({NSSI thought}_{ti} = 1 \vert{Emptiness}_{ti})}= \frac{1}{{1+e}^{-(\alpha_{6i}+\beta_{6i}{emp}_{ti})}}$ |

${NSSI thought}_{ti}$ is the occurrence of the NSSI thought for the *t*-th report in *i*-th participant. The subscript *ti* for the predictors also represents the values of the predictors for the t-th report in the i-th participant.

**Between-level**

| $\beta_{0i}=\gamma_{00}+ u_{0i}$ |  |
| --- | --- |
| $\beta_{1i}=\gamma_{10}+u_{1i}$ |  |
| $\beta_{2i}=\gamma_{20}+ u_{2i}$ |  |
| $\beta_{3i}=\gamma_{30}+ u_{3i}$ |  |
| $\beta_{4i}=\gamma_{40}+ u_{4i}$ |  |
| $\beta_{5i}=\gamma_{50}+ u_{5i}$ |  |
| $\beta_{6i}=\gamma_{60}+ u_{6i}$ |  |
| $\left[ u_{0i} u_{1i} u_{2i} u_{3i} u_{4i} u_{5i} u_{6i} \right]\sim N\left( \left[ 0 0 0 0 0 0 0 \right],\left[ \tau_{00} \tau_{10} \tau_{20} \tau_{30} \tau_{40} \tau_{50} \tau_{60} \right] \right)$ |  |

The subscript 'i' in the between-level variables represents the slope of the i-th person for each variable. $\gamma_{00}, \gamma_{01}, \gamma_{02}, \gamma_{03}, \gamma_{04}, \gamma_{05}, \gamma_{06,}$ are the fixed effect of the predictors. The vector of random effects, $u_{0i}, u_{1i}, u_{2i}, u_{3i}, u_{4i}, u_{5i}, u_{6i,}$ are assumed to follow multivariate normal distribution with mean vector 0 and variance $\tau_{00}, \tau_{01}, \tau_{02}, \tau_{03}, \tau_{04}, \tau_{05}, \tau_{06}$.

**Appendix 2. Examples of the EMA questionnaires used in this study**

**Self-Injury and Suicidality Questionnaire (English Version)**

**Section A. Thoughts of Self-Injury and Suicide**

1. Since the las assessment, have you ever thought about injuring yourself of attempting suicide?

(Please mark the appropriate box.)

| **Item** |  | **Yes** | **No** |
| --- | --- | --- | --- |
| 1-1 | Self-injury |  |  |
| 1-2 | Suicide attempt |  |  |

*In the app, if a participant selected “Yes” to either item, the program automatically displayed Q2; if the participant selected “No” to both, the program skipped to Q5.*

**Section B. Self-Injurious Behavior**

2. Since the last assessment, have you actually engaged in any behavior that harmed yourself?

 Yes

 No

*In the app, if a participant selected “Yes” on Q2, the program automatically displayed Q3, which was followed by Q4; if the participant selected “No” on Q2, the program skipped to Q5.*

3. When engaging in self-injurious behavior since the last assessment, did you think about suicide or wish you were dead immediately before or during the act?

(Please mark the statement that best applies.)

 Not at all.

 I thought briefly about death but did not take it seriously.

 I thought about death more seriously, and at that time I wanted to die.

 I thought very seriously about suicide and wanted to die.

**Section C. Methods of Self-Injury**

4. Since the last assessment, which of the following methods have you used to harm yourself? (Check all that apply.)

 Cutting or scratching the body with a sharp object.

 Hitting of punching the body (e.g., punching a wall, hitting one’s head).

 Excessive use of alcohol or drugs.

 Burning, picking, or otherwise damaging the skin.

 Piercing or punching the body with a sharp object.

 Other (please specify): ______________________

**Mood Appraisal Questionnaire (English Version)**

5. Please read each statement carefully and select the number on the scale that best reflects the degree to which it applies to you.

| **Item** | **Question** |
| --- | --- |
| 5-1 | “How depressed do you feel right now?” |
| 5-2 | “How sad do you feel right now?” |
| 5-3 | “How anxious do you feel right now?” |
| 5-4 | “How fearful do you feel right now?” |
| 5-5 | “How lonely do you feel right now?” |
| 5-6 | “How rejected or hurt do you feel right now?” |
| 5-7 | “How angry at yourself do you feel right now?” |
| 5-8 | “How angry at others do you feel right now?” |
| 5-9 | “How ashamed do you feel right now?” |
| 5-10 | “How empty do you feel right now?” |

**Response scale for all items:**

| 0 | 1 | 2 | 3 | 4 | 5 | 6 | 7 | 8 |
| --- | --- | --- | --- | --- | --- | --- | --- | --- |
| Not at all |  | Slightly |  | Moderately |  | Quite a bit |  | Extremely |

*In the app, responses were captured on this 0-8 slider with the above verbal anchors.*
